# Correlation between the TiO_2_ encapsulation layer on Pt and its electrochemical behavior†

**DOI:** 10.1039/d1na00423a

**Published:** 2021-07-15

**Authors:** Raquel Aymerich Armengol, Joohyun Lim, Marc Ledendecker, Katharina Hengge, Christina Scheu

**Affiliations:** Max-Planck Institut für Eisenforschung GmbH Max-Planck-Straße 1 40237 Germany c.scheu@mpie.de; Department of Chemistry, Kangwon National University Chuncheon Gangwon 24341 Republic of Korea jlim@kangwon.ac.kr; Department of Technical Chemistry I, Technical University Darmstadt Alarich-Weiss-Straße 8 64287 Germany

## Abstract

Supported metal catalysts with partial encapsulation resulting from strong metal–support interactions show distinctive structural features which strongly affect their functionalities. Yet, challenges in systematic synthesis and in-depth characterization for such systems limit the present understanding of structure–property relationships. Herein, the synthesis and characterization of two Pt/TiO_2_ models are conducted by a simple change of the synthesis order, while keeping all other parameters constant. They differ in containing either bare or encapsulated Pt nanoparticles. The presence of an extremely thin and inhomogeneous TiO_2_ layer is clearly demonstrated on 2–3 nm sized Pt nanoparticles by combination of imaging, energy dispersive X-ray spectroscopy and electron energy loss spectroscopy performed in a transmission electron microscope. The two Pt/TiO_2_ systems exhibit differences in morphology and local structure which can be correlated with their electrochemical activity and stability using cyclic voltammetry experiments. Beyond enhanced particle stability, we report an increase in H^+^ intercalation on titania and reduced Pt activity due to partial encapsulation by TiO_2_. Finally, the growth of an encapsulation layer as a result of cyclic voltammetry measurements is discussed. These results shed light on the in-depth structure–property relationship of catalysts with strong metal–support interactions which leads to enhanced functional materials for electrochromic devices and energy applications.

## Abstract 2

Partial TiO_2_ encapsulation of Pt nanoparticles on TiO_2_ support due to strong metal–support interactions is revealed by TEM and correlated to the electrochemical properties. Changes related to multiple electrochemical cycling are investigated.

## Graphical abstract

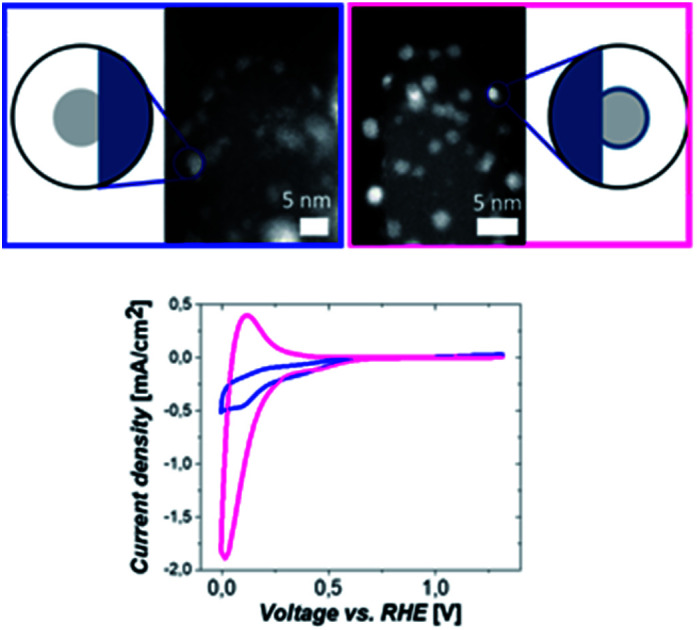

## 1. Introduction

The properties of supported catalysts depend on the local structure and interaction between the catalyst and the support. A particular example is materials showing strong metal–support interactions (SMSI). The electronic and structural modifications of SMSI yield high stability against agglomeration as well as enhanced activity and selectivity.^1–3^

SMSI can generally be achieved through annealing of metal catalysts supported by reducible metal oxides such as TiO_2_, WO_3_ or Nb_2_O_5_ in a reducing atmosphere.^4,5^ During the annealing process, atoms of the metal oxide support can diffuse and cover the metal catalyst forming an encapsulation layer. Thin and/or partial encapsulation of the catalyst by the support material was described to be responsible for extraordinary stability, activity and selectivity in various metal/metal oxide systems.^6,7^ Yet, impermeable and thick support layers over the catalysts can also lead to a decrease in performance by blocking active sites.^8,9^ Therefore, probing the existence and specific role of the encapsulation layer of SMSI materials is crucial to understanding their performance in catalytic reactions. Although the existence of thin encapsulation layers on metal catalysts was recently proved by electron microscopy,^10–14^ structure–property relationships were rarely investigated, particularly in the case of electrochemical properties.^15–17^ The lack of systematic studies is related to both challenges in the synthesis and characterization of comparable model materials with and without an encapsulation layer. Moreover, the high temperature and reducing atmosphere employed to obtain SMSI materials often cause sintering and perfect encapsulation of the catalyst instead of thin and/or inhomogeneous encapsulation layers.^18–20^

Among various supported metal systems, Pt/TiO_2_ is recognized as a promising material for electrochromic devices^21^ and as an anode in fuel cells where it is investigated as a substitute for platinum nanoparticles supported on carbon (Pt/C) due to its superior corrosion stability.^22,23^

Furthermore, a very recent study reported that encapsulated Pt on TiO_2_ can act as a selective catalyst for the hydrogen oxidation reaction.^16^ On the one hand, the selective H^+^ permeability through the TiO_2_ encapsulation layer prevents the oxygen reduction reaction on Pt. On the other hand, selectivity can also be driven by exploiting the conductivity dependence of the material. This depends on the mixed valences of Ti and the reversible formation of conductive H_*x*_TiO_2_ or insulating TiO_2_ under H_2_ and O_2_ atmospheres, respectively.^24^

In the present study, we aim to determine the effect of SMSI encapsulation layers on the electrochemical behavior of Pt/TiO_2_ nanomaterials. For this purpose, we synthesize and analyze Pt nanoparticles on TiO_2_ nanowires with and without a TiO_2_ encapsulation layer and compared their performance and stability. A combination of (scanning) transmission electron microscopy ((S)TEM), energy-dispersive X-ray spectroscopy (EDS) and electron energy loss spectroscopy (EELS) techniques is used to probe the presence of an extremely thin encapsulation layer. The structure of the materials is correlated to the electrochemical response and stability by cyclic voltammetry (CV). Our results determine the precise structure of a supported metal catalyst material and reveal how an extremely thin encapsulation layer affects its electrochemical behavior. Such an encapsulation layer can form directly during synthesis or during the CV experiments. The findings are essential for understanding structure–property relationships for electrocatalysts showing SMSI.

## 2. Synthesis of Pt/TiO_2_

**Fig. 1:**
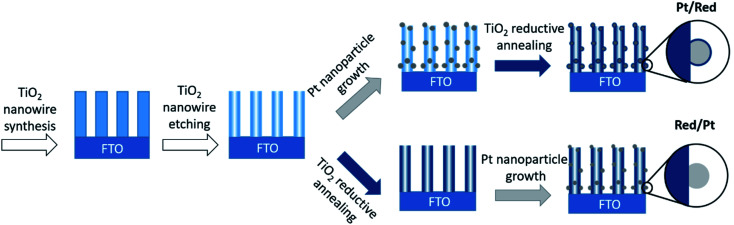
Schematic drawings of the synthesis of two Pt/TiO_2_ systems. Note that only the order of the synthesis steps was changed.

Pt nanoparticles on TiO_2_ nanowires were synthesized by two different chemical routes (Fig. 1). In both cases, TiO_2_ nanowires were grown on a fluorine-doped tin oxide (FTO) electrode through a hydrothermal method and etched to a hollow structure with a high specific surface area.^25,26^ Divalent tin cations (Sn^2+^) were adsorbed to the surface oxyanions and hydroxyl groups, which served as nucleation points for the reduction of the Pt precursor to Pt nanoparticles.^27^

To obtain the first Pt/TiO_2_ model with a TiO_2_ encapsulation layer on Pt nanoparticles (denoted as Pt/Red), Pt nanoparticles were grown on the surface of hollow TiO_2_ nanowires and the entire Pt/TiO_2_ composite was subsequently annealed in the presence of sodium borohydride as a reducing agent under a nitrogen atmosphere. The reduction process induces the formation of a TiO_2_ encapsulation layer on Pt nanoparticles^4^ and increases the conductivity of the titania support by introducing oxygen vacancies and trace impurities.^28,29^ To obtain the second Pt/TiO_2_ model without an encapsulation layer (denoted as Red/Pt), hollow TiO_2_ nanowires were reduced before the growth of Pt nanoparticles. This different reaction order allows us to synthesize two distinct Pt/TiO_2_ materials to study the TiO_2_ encapsulation layer on Pt, while fixing most other variables (Fig. 1).

**Fig. 2:**
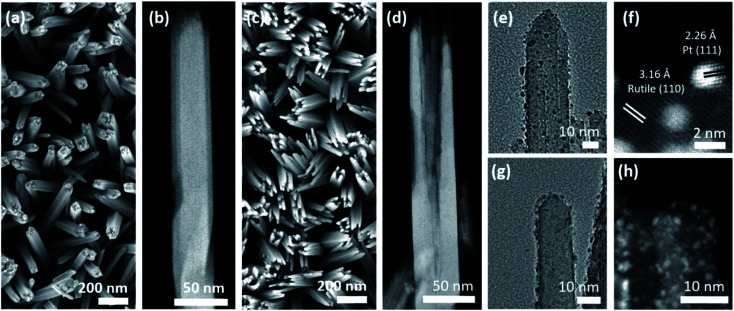
Top-view SEM and side-view HAADF-STEM micrographs of the as-synthesized TiO_2_ nanowire array (a and b) before and (c and d) after etching. Side-view TEM and HAADF-STEM micrographs for a single nanobundle of (e and f) Pt/Red and (g and h) Red/Pt. Please note that (f) and (h) were taken at different regions compared to (e) and (g).

The size, morphology, and crystal structure of Pt/TiO_2_ were verified by electron microscopy (Fig. 2). Scanning electron microscopy (SEM) shows TiO_2_ nanowires grown on a FTO electrode in the top-view (Fig. 2a), while the high angle annular dark-field (HAADF)-STEM image in Fig. 2b displays a single detached nanowire composed of smaller nanobundles in the side-view. After etching the inner part of TiO_2_ nanowires, only the external nanobundles remain (Fig. 2c and d). This yields hollow TiO_2_ nanowires with a larger surface area to accommodate a larger amount of Pt nanoparticles.

TEM and HAADF-STEM images display Pt nanoparticles in black and white, respectively, for both Pt/Red (Fig. 2e and f) and Red/Pt (Fig. 2g and h). Pt nanoparticles are homogeneously distributed over the TiO_2_ surface (see ESI movie1† for the 3D reconstructed volume from a single nanobundle of Pt/Red using electron tomography). The high magnification STEM image in Fig. 2f reveals the (111) lattice planes of Pt nanoparticles and the (110) lattice planes of rutile TiO_2_.

## 3. Particle size and catalyst loading

**Fig. 3:**
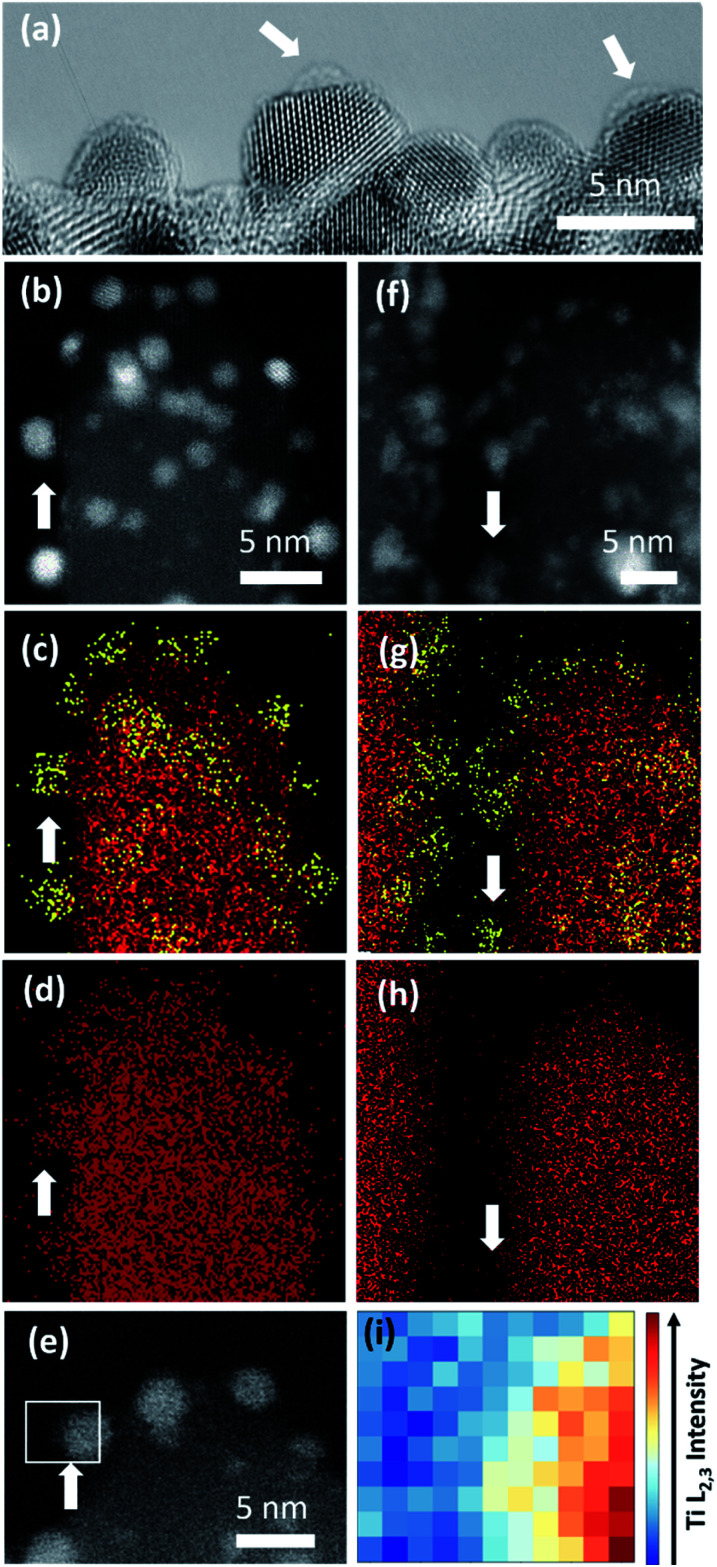
(a) HRTEM image of Pt nanoparticles of Pt/Red with a visible TiO_2_ encapsulation layer (indicated with white arrows). In the other Pt surface parts the layer is <5 Å which would correspond to 2 (111)_Pt_ atomic layers. HAADF-STEM image and EDS maps of (b–d) Pt/Red and (f–h) Red/Pt. Ti and Pt are denoted in red and yellow colors, respectively. White arrows indicate the areas where Pt is exposed out of a single TiO_2_ nanobundle. (e) HAADF-STEM image of Pt/Red with the white square indicating a Pt nanoparticle on the surface above the vacuum where EELS analysis in (i) was performed. (i) Intensity distribution of the EELS Ti L_2,3_-edge from region marked in (e).

A larger average size of Pt particles in Pt/Red (2.4 ± 0.6 nm) than that of Red/Pt (1.6 ± 0.4 nm) was found (Fig. S1†). The encapsulation layer in Pt/Red is barely visible in HRTEM images (Fig. 3a). From the obtained micrographs, one can conclude that the average thickness of the encapsulation layer is below 1–2 (111)_Pt_ atomic layers. We believe that this layer is not from any contaminations including carbon based on our careful sample preparation which included plasma cleaning.

The presence of such a thin TiO_2_ layer seems to efficiently prevent the growth of Pt nanoparticles at high temperature^30^ since the average size of Pt nanoparticles in Pt/Red is not distinguishable from the particle size before the reductive annealing (2.5 ± 0.4 nm, Fig. S1†). The stability of nanoparticles at high temperatures is beneficial for catalysis, yet it is rarely reported even for SMSI systems.^18,19^

Inductively coupled plasma mass spectroscopy (ICP-MS) determined a higher Pt concentration per electrode area for Pt/Red (2.85 ± 0.08 μg cm^−2^) than that for Red/Pt (0.37 ± 0.01 μg cm^−2^).

We hypothesize that the difference in the size of the Pt nanoparticles and Pt loading is related to a different number of surface adsorption sites on the TiO_2_ substrates. In the synthetic approach employed to deposit Pt nanoparticles,^27^ Sn^2+^ cations are adsorbed onto oxyanions and hydroxyl groups of TiO_2_ by electrostatic interactions. The Sn^2+^ cations reduce Pt^4+^ and the yielded Pt^0^ nanoparticles remain adsorbed at the surface sites while Sn^4+^ cations are dissolved in the solution. The Pt nanoparticles are able to grow at these sites. However, in Red/Pt the oxyanions and hydroxyl groups are partially removed during heat treatment before deposition of the Pt. Thus, fewer growth sites for Pt are available in the Red/Pt system, resulting in lower Pt loading and smaller nanoparticles than those of Pt/Red.

## 4. Spectroscopy analysis of the encapsulation layer

To further characterize the TiO_2_ encapsulation layer on Pt nanoparticles, both Pt/TiO_2_ systems were analyzed using spectroscopy techniques (Fig. 3). EDS maps obtained in STEM mode display Ti (red color) distributed around the Pt nanoparticles (yellow color) of Pt/Red (Fig. 3b–d). This Ti corresponds to the TiO_2_ encapsulation layer grown during the reductive annealing of Pt on the TiO_2_ support. In contrast, the EDS of Red/Pt does not reveal a Ti layer covering the Pt nanoparticles (Fig. 3f–h) and no visible encapsulation layer was found by HRTEM analyses (Fig. S2†).

To corroborate our results with shorter electron beam exposure and higher spatial resolution, EELS was conducted in STEM mode. Representative spectra were measured on encapsulated Pt nanoparticles lying on the surface of TiO_2_ nanobundles to prevent contributions from the preexisting TiO_2_ (see Fig. 3e). Fig. 3i displays the intensity distribution of the Ti L_2,3_-edge over the Pt/Red Pt nanoparticle indicated in Fig. 3e. The visualized Ti signal implies the formation of a TiO_2_ encapsulation layer surrounding the Pt nanoparticle on Pt/Red, while no Ti coverage was found for Red/Pt (Fig. S3†). The Ti signal intensity is higher at the Pt/TiO_2_ interface and spreads inhomogeneously throughout the Pt nanoparticle, while fading completely at the edge of the nanoparticle (see Fig. S4†). This suggests a partial TiO_2_ encapsulation on the Pt nanoparticles. An “incomplete” encapsulation is desirable for highly stable and selective catalysts without losing activity during application.

## 5. Electrochemical behavior

**Fig. 4:**
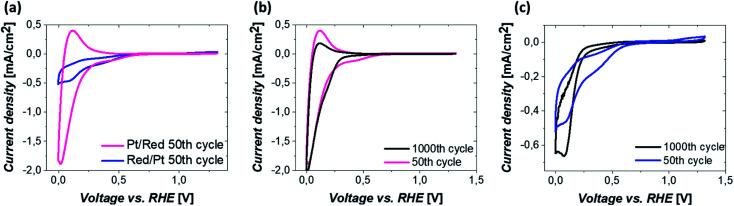
(a) CVs after 50 CV cycles of Pt/Red and Red/Pt. Comparison of CVs after 50 and 1000 CV cycles of (b) Pt/Red and (c) Red/Pt. All measurements were conducted under an oxygen atmosphere in 0.1 M HClO_4_ electrolyte at a scan rate of 200 mV s^−1^.

The electrochemical behavior of the two Pt/TiO_2_ systems was compared using CV by scanning from 0 to 1.2 V_RHE_ at a scan rate of 200 mV s^−1^ in an oxygen saturated 0.1 M perchloric acid (HClO_4_) electrolyte. The results after stabilization of the electrodes (see Fig. S5†) can be found in Fig. 4a. The majority of the electrochemical response of the electrodes in the range 0–0.2 V_RHE_ arises from the hollow TiO_2_ nanowires. Nevertheless, contributions of Pt can also be observed in both samples despite the low Pt loading.

The major CV feature of Pt/Red after 50 cycles (Pt/Red_50CV_) is the broad anodic peak at *ca.* 0.1 V_RHE_ and its corresponding cathodic peak at *ca.* 0.0 V_RHE_. These peaks can be attributed to both hydrogen underpotential deposition (H_UPD_) on Pt and H^+^ intercalation on TiO_2_ leading to H_*x*_TiO_2_:^31–33^1
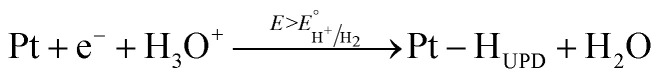
2
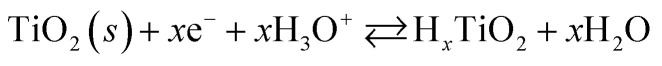


The overlap between H_UPD_ and H^+^ intercalation hinders a detailed understanding of the exact contribution of each reaction to the overall observed charge and limits a straightforward evaluation of the electrochemically active surface area.^34^ Yet, the single broad peak in the range of 0 and 0.1 V_RHE_ implies that the H^+^ intercalation in TiO_2_ is a major contributor for Pt/Red_50cv_. In contrast, the 50th cycle of Red/Pt (named Red/Pt_50cv_) shows a markedly different peak shape and current density compared to Pt/Red_50cv_ with one cathodic maximum at 0.1 V_RHE_ and one anodic maximum at 0.2 V_RHE_. Such a difference suggests a relatively low H^+^ intercalation to H_UPD_ contribution compared to Pt/Red.

The reason behind the enhanced contribution of H^+^ intercalation in the encapsulated Pt/Red system compared to Red/Pt is hydrogen spillover. This consists in the migration of adsorbed H on Pt towards TiO_2_, aiding the formation of the H_*x*_TiO_2_ phase.^35–38^ Furthermore, hydrogen spillover is particularly boosted in materials with SMSI due to the presence of a larger metal–metal oxide interface^15^ which explains the increased H^+^ intercalation in Pt/Red.

The two Pt/TiO_2_ systems exhibit a cathodic peak at *ca.* 0.4 V_RHE_ after the 50th cycle. This feature can be attributed to the presence of Pt (H_UPD_ or Pt reduction, which can take place in a broad potential range due to the H^+^ intercalation on TiO_2_ (ref. 39)). The oxygen reduction reaction (ORR) also takes place in both systems^33^ but is more remarkable in Red/Pt_50CV_, visibly shifting the H^+^ intercalation and H_UPD_ peaks towards negative current densities. Moreover, only Red/Pt_50CV_ shows an additional peak at 1.2 V_RHE_ which can be assigned to the onset of Pt oxidation. This suggests a higher accessibility of Pt-related reactions for Red/Pt than for Pt/Red despite its lower Pt content as determined by ICP-MS. Clearly, Pt/Red possesses a lower number of exposed Pt active sites due to the partial TiO_2_ encapsulation of Pt nanoparticles.

Our results imply that the electrochemical behavior of Pt/TiO_2_ can be finely controlled by extremely thin encapsulation layers on the platinum surface. Because of the enhanced H^+^ intercalation, Pt/Red can be a promising material in electrochromic applications such as smart displays.^40,41^ Furthermore, the encapsulating layer can be used for catalytic reactions with higher selectivity for fuel cell applications.^16,42^

To investigate the electrochemical stability of both Pt/TiO_2_ systems, 1000 CV measurements between 0 and 1.2 V_RHE_ were conducted under oxygen saturation conditions and compared with the 50th cycle. The anodic peak of Pt/Red after 1000 cycles at 0.1 V_RHE_ reduces in intensity, while the corresponding cathodic peak slightly increases (Pt/Red_1000CV_, Fig. 4b). This might be the consequence of a growth in thickness and Pt surface coverage of the TiO_2_ encapsulation layer, which further enhances the H_*x*_TiO_2_ phase formation. In contrast, for 1000 times cycled Red/Pt (Red/Pt_1000CV_, Fig. 4c), the shape of the CV peaks attributed to H_UPD_ and H^+^ intercalation changed and the anodic peak at 0.2 V almost disappeared. This behavior could be explained by the formation of an encapsulation layer also for Red/Pt during cycling even at room temperature.^17^ In such a case, due to the gradual encapsulation, the contribution of H^+^ intercalation in Red/Pt increases yielding a broader peak in the range of 0–0.25 V_RHE_ for Red/Pt_1000CV_ which resembles that of Pt/Red_50CV_. During cycling, the Pt oxidation peak at 1.2 V_RHE_ disappears, as well as the peak at 0.4 V_RHE_. These changes indicate the loss of Pt active sites either as a consequence of catalyst degradation or due to the growth of TiO_2_ over Pt nanoparticles during electrochemical redox cycling.^17^

**Fig. 5:**
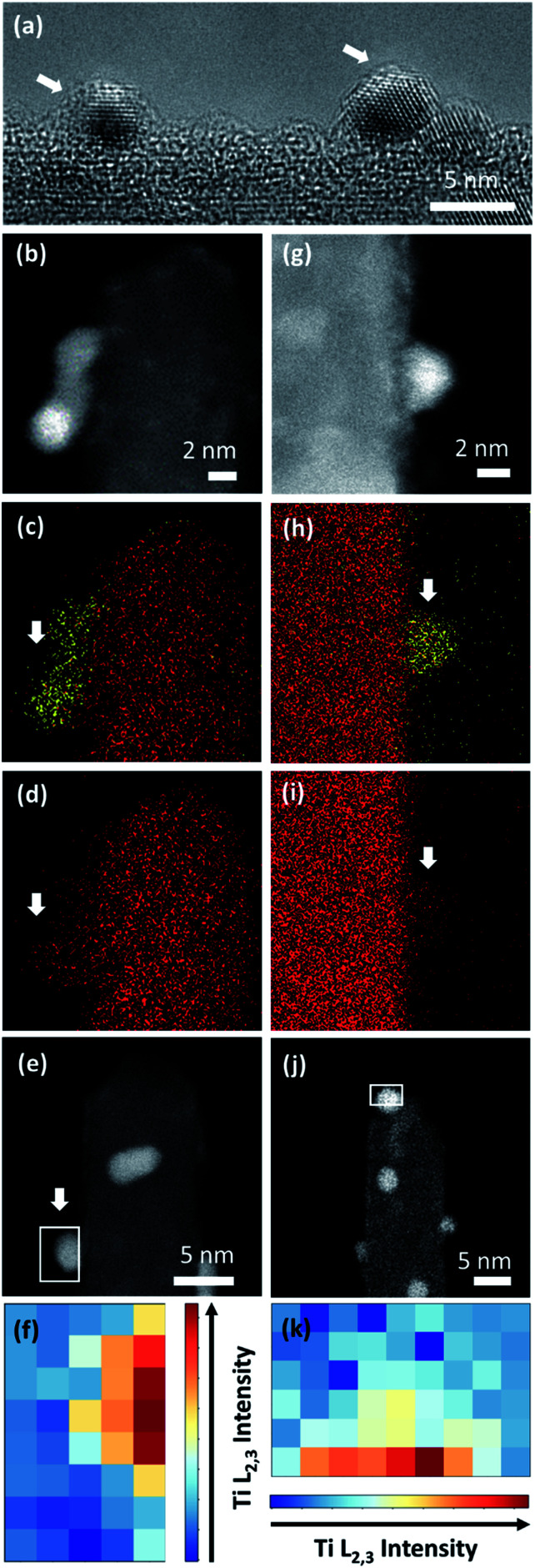
(a) HRTEM image of Pt nanoparticles of Pt/Red after 1000 CV cycles with a visible TiO_2_ encapsulation layer (indicated with white arrows). HAADF-STEM image and EDS maps of (b–d) Pt/Red and (h–i) Red/Pt after 1000 CV cycles. Ti and Pt are denoted with red and yellow colors, respectively. White arrows indicate the areas where Pt is exposed to vacuum and attached to a single TiO_2_ nanobundle. HAADF-STEM image after 1000 CV cycles of Pt/Red (e) and Red/Pt (j) indicating the area where EELS analysis was performed. (f and k) Intensity distribution of EELS Ti L_2,3_ core loss on a Pt nanoparticle after 1000 CV cycles for Pt/Red and Red/Pt, respectively.

To confirm the CV-driven encapsulation of Pt nanoparticles and further structural changes in the employed potential region, the local structure of the two Pt/TiO_2_ systems was analyzed after 1000 CV cycles (Fig. 5). HRTEM analyses (Fig. 5a) and EDS maps (Fig. 5b–d) confirmed the presence of encapsulated Pt nanoparticles on Pt/Red_1000CV_. Notably, EDS analyses for Red/Pt_1000CV_ also show a low Ti signal at the Pt nanoparticles (Fig. 5g and j). EELS analyses confirmed the existence of a TiO_2_ layer covering the Pt nanoparticles after cycling for both samples. HAADF images and the intensity distribution of the Ti L_2,3_-edges of selected particles are shown in Fig. 5e and f for Pt/Red_1000CV_ and in Fig. 5j and k for Red/Pt_1000CV_, respectively. The presence of a Ti signal observed by EDS and EELS in Red/Pt_1000CV_, which was not observed for the initial material, strongly supports the growth of an encapsulation layer as a result of electrochemical cycling. A Ti L_2,3_-edge signal is, however, not present for all Pt nanoparticles in the Red/Pt system, suggesting the presence of both encapsulated and uncovered Pt nanoparticles after 1000 CV cycles. This is consistent with the observed voltammogram for Red/Pt_1000CV_ (Fig. 4c), which shows intermediate behavior between encapsulated (Pt/Red_50CV_, Fig. 4b) and encapsulation-free models (Red/Pt_50CV_, Fig. 4c).

Pt nanoparticle degradation might also contribute to the electrochemical changes observed prior to and after electrochemical cycling. Fig. 5b and g show Pt nanoparticles for the Pt/Red_1000CV_ and Red/Pt_1000CV_, respectively. The average nanoparticle size of Red/Pt increased from 1.6 ± 0.4 to 3.3 ± 0.7 nm (relative size increase of 100%) with cycling (Fig. S1c and e†). For Red/Pt, the encapsulation of a few Pt nanoparticles by TiO_2_ does not prevent an average growth in the relative particle size during cycling. In the case of Pt/Red, the nanoparticles grew from 2.4 ± 0.6 to 3.0 ± 0.6 nm (relative size increase of ∼25%) (Fig. S1b and d†). This demonstrates that for Pt/Red, the presence of an encapsulation layer clearly increases the stability and prevents agglomeration. Our results highlight the protective role of the initial thin encapsulation layer against particle aggregation during the employed electrochemical degradation cycles.^19,43–45^

**Fig. 6:**
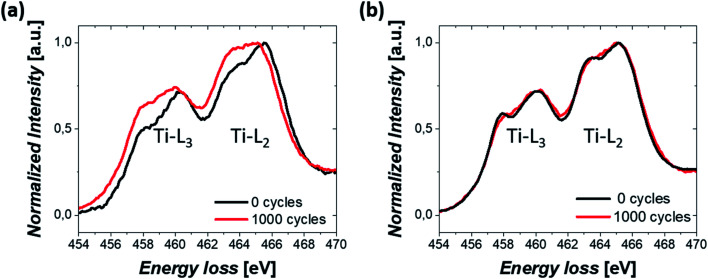
EELS spectrum showing Ti-L_2,3_ white lines from a nanobundle of (a) Pt/Red and (b) Red/Pt samples before and after 1000 CV cycles.

Finally, the change in the oxidation state of Ti was investigated. TiO_2_ was initially partially reduced to increase the conductivity of the Pt/TiO_2_ material system through the reduction step.^46^ Therefore, the stability of the oxidation state of Ti during electrochemical cycles could also modify the properties of Pt/TiO_2_ materials. EELS mapping was conducted on TiO_2_ nanobundles prior to and after 1000 CV cycles and the averaged spectra over the whole area are shown in Fig. 6 for both Pt/Red and Red/Pt. Both spectra show Ti L_2,3_ white lines and similar energy losses for the uncycled samples (see Fig. S6†). However, for Pt/Red, the peaks show lower splitting and are shifted towards lower energy losses by *ca.* 0.4 eV after 1000 cycles (Fig. 6a). This indicates a slight reduction of the TiO_2_ support upon electrochemical cycling. Such a reduction is a consequence of the enhanced H^+^ intercalation in Pt/Red which is not fully reversible.^16^ In contrast, Red/Pt displays similar energy loss of the Ti L_2,3_ edge before and after electrochemical cycling (Fig. 6b), indicating a higher stability of the TiO_2_ support with no or only slight reduction. This can be attributed either to lower H^+^ intercalation and/or higher reversibility of such a reaction for Red/Pt.

In both samples, the lack of re-oxidation of the partially reduced TiO_2_ after electrochemical cycling underlines its high stability, a promising characteristic for future application of Pt/TiO_2_ materials.

## 6. Conclusions

Two Pt/TiO_2_ systems consisting of Pt nanoparticles on partially reduced TiO_2_ nanowires were synthesized with and without partial TiO_2_ encapsulation of the Pt surface. The presence of an inhomogeneous TiO_2_ encapsulation layer thinner than 1–2 (111)_Pt_ layers was confirmed on Pt nanoparticles (<3 nm) by a combination of HRTEM, EDS and EELS. The encapsulated nanoparticles possess higher stability against agglomeration at high temperatures and in electrochemical measurements. We could also correlate the partial encapsulation of Pt with an enhanced and partially irreversible H^+^ intercalation, explained by enhanced hydrogen spillover on the encapsulated Pt/TiO_2_, as well as reduced Pt activity. Moreover, our results also demonstrate the formation of nanoparticle encapsulation caused by electrochemical cycling, thus modifying the long-term electrochemical behavior and application of the materials.

## 7. Experimental section

### 7.1 Materials

Titanium(iv) butoxide (Ti(OC_4_H_9_)_4_), hydrochloric acid (HCl, 37 wt%), hydrogen hexachloroplatinate solution (H_2_PtCl_6_, ∼8 wt% in H_2_O), sodium formate (HCOONa), tin(ii) chloride dihydrate (SnCl_2_·2H_2_O), sodium borohydride (NaBH_4_) and fluorine-doped tin oxide glass (F:SnO_2_, FTO) were purchased from Sigma Aldrich. All reagents were used without further purification.

### 7.2 Synthesis

#### 7.2.1 Growth of titanium dioxide nanowires

TiO_2_ nanowires were synthesized on FTO through a hydrothermal synthesis protocol.^26^ First, the FTO electrodes were cleaned with acetone, ethanol, propanol and water. Then, two electrodes were placed in a Teflon liner with a HCl solution (10 ml, 6.1 M) and titanium butoxide (200 μl). The Teflon liner was sealed in an autoclave and heated in an oven at 180 °C for 2 h. After cooling down to room temperature, the electrodes were rinsed with deionized water and dried in air.

#### 7.2.2 Etching of titanium dioxide nanowires

Two FTO electrodes covered by TiO_2_ nanowires were again placed in a Teflon liner with a HCl solution (10 ml, 6.1 M) and heated inside an autoclave at 180 °C for 2 h.^25^ After cooling down to room temperature, the electrodes were rinsed with deionized water and dried in air.

#### 7.2.3 Synthesis of Pt/Red and Red/Pt

To synthesize Pt/Red samples, the growth of Pt nanoparticles was conducted on TiO_2_ hollow nanowires and the resulting Pt/TiO_2_ nanostructure was subsequently reduced. For Red/Pt the hollow TiO_2_ nanowires were first reduced and then Pt nanoparticles were grown on them (Fig. 1). In both cases, identical protocols were used to grow Pt nanoparticles and to reduce the TiO_2_ support, respectively:

##### Growth of Pt nanoparticles

Two TiO_2_ electrodes (for Pt/Red: without reduction, for Red/Pt: after reduction) were placed inside a beaker. Deionized water (10 ml), SnCl_2_·2H_2_O (20 mg) and HCl (20 μl) were added in this order. The beaker was sonicated (*ca.* 10 s) in an ultrasound bath and stirred for 10 min. The electrodes were then rinsed with HCl solution (0.2 M) and soaked in H_2_PtCl_6_ solution (10 ml, 0.2 mM). Pt nanoparticles were grown by dropwise addition of HCOONa solution (5 ml, 4 mM) and stirring for 5 h.^27^

##### Reductive annealing

Two (Pt/)TiO_2_ electrodes were introduced in a crucible over NaBH_4_, with the glass side contacting the chemical powder. The crucible was heated under nitrogen flow in a tubular furnace at 400 °C for 2 h.^47^ After cooling down to room temperature, the electrodes were removed from the furnace and rinsed with deionized water to eliminate excess NaBH_4_.

### 7.3 Characterization

#### 7.3.1 Scanning electron microscopy (SEM)

Scanning electron microscopy was used to investigate the morphology. A Zeiss Gemini SEM was operated with an acceleration voltage of 1.5 kV and images were acquired with the in-lens secondary electron detector.

#### 7.3.2 Transmission electron microscopy (TEM) and scanning transmission electron microscopy (STEM)

Measurements were done at 300 kV using two ThermoScientific Titan Themis S/TEM instruments, one equipped with a C_S_ image corrector and one with a C_S_ probe corrector. Imaging was performed either in HRTEM mode or using the attached HAADF-STEM detector to characterize morphologies, particle sizes and lattice planes.

#### 7.3.3 Energy-dispersive X-ray spectroscopy (EDS) and electron energy loss spectroscopy (EELS)

Mapping was performed in a probe corrected Titan Themis S/TEM using the attached Bruker Super X-EDX detector and the Gatan Quantum ERS energy filter, respectively.

#### 7.3.4 Inductively coupled plasma mass-spectroscopy (ICP-MS)

The platinum loading of the samples was measured using a NexION 300 PerkinElmer ICP-MS. The results correspond to the average of two sets per sample type. The given uncertainty interval is the higher value between the statistical uncertainty due to averaging and the precision of the technique.

#### 7.3.5 Cyclic voltammograms (CV)

Measurements were performed in an electrochemical Teflon cell with Pt/TiO_2_ as the working electrode, Ag/AgCl (3 M KCl) as the reference electrode and a graphite counter electrode. Typically, the applied potentials were in the −0.22 to 1.10 V range *vs.* Ag/AgCl with a scan rate of 200 mV s^−1^. The electrolyte used was 0.1 M HClO_4_. The current has been normalized to the geometric surface area of the Pt/TiO_2_/FTO electrodes (*ca.* 1 cm^2^).

## Conflicts of interest

There are no conflicts to declare.

## Supplementary Material

NA-003-D1NA00423A-s001

NA-003-D1NA00423A-s002
