# Supplementary material for: Correlation between the TiO2 encapsulation layer on Pt and its electrochemical behavior
Source: Nanoscale Adv. 2021 Jul 15;3(17):5075–82. doi: 10.1039/d1na00423a (PMC9417513; doi:10.1039/d1na00423a)
Supplement: NA-003-D1NA00423A-s002 [file NA-003-D1NA00423A-s002.pdf]

Electronic Supplementary Material (ESI) for Nanoscale Advances.
This journal is © The Royal Society of Chemistry 2021

Supporting Information

Direct correlation between encapsulation layer on Pt/TiO_2 and electrochemical behavior

Raquel Aymerich Armengol, Joohyun Lim, Marc Ledendecker, Katharina Hengge, and Christina Scheu**

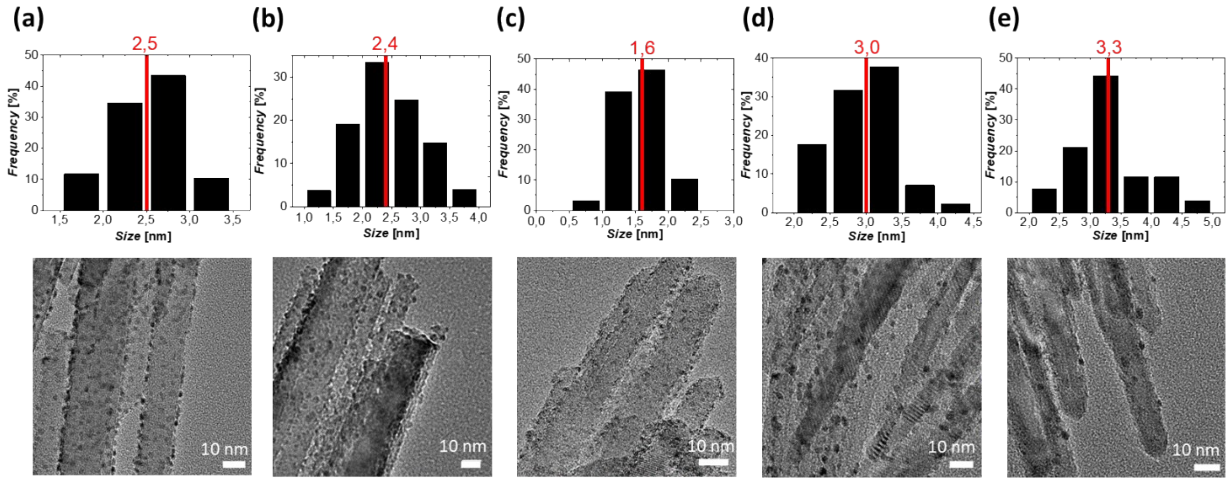

Figure S1. Size distribution of Pt nanoparticles and representative TEM images for (a) Pt/TiO_2 without reduction, (b) Pt/Red, (c) Red/Pt, (d) Pt/Red after 1000th CV and (e) Red/Pt after 1000th CV. Red lines indicate the mean nanoparticle size.

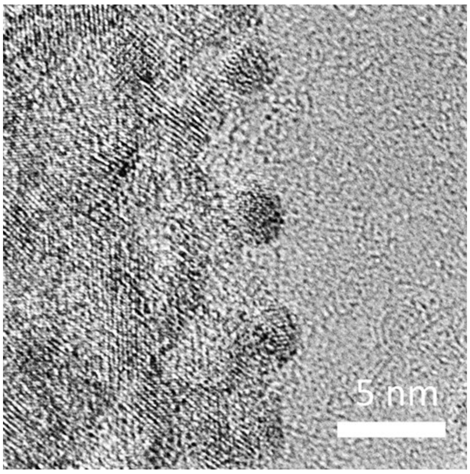

Figure S2. HRTEM micrograph of Red/Pt. Note that the background in the right shows the C support of the TEM grid.

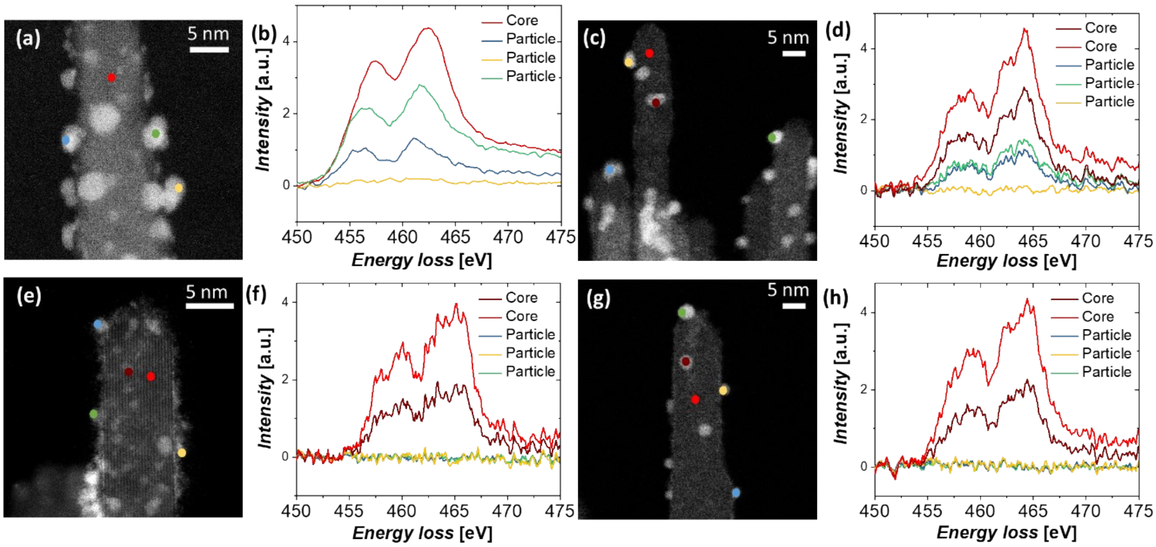

Figure S3. HAADF micrograph revealing the location of the EELS point analyses and corresponding EELS spectra with (a) and (b) for Pt/Red before CV, (c) and (d) for Pt/Red after 1000 CV, (e) and (f) for Red/Pt before CV and (g) and (h) for Red/Pt after 1000 CV.

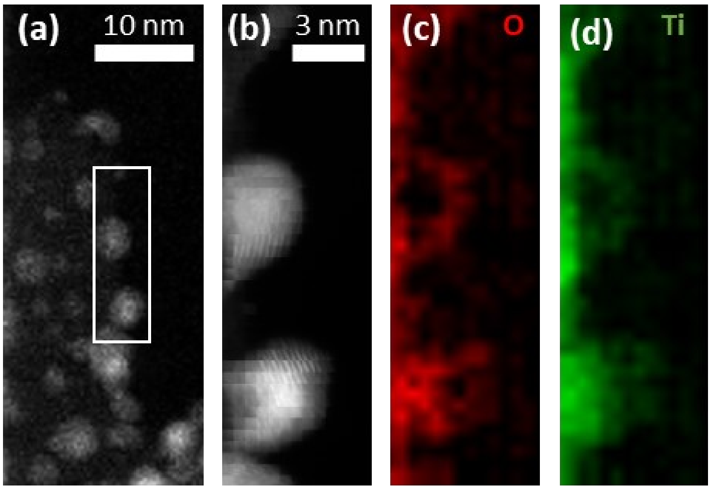

Figure S4. (a) HAADF image of a Pt/Red nanobundle. The squared region, corresponding to the edge of the nanobundle with two platinum nanoparticles, is analyzed by EELS. (b) Zoom in HAADF image of the area marked in (a). (c) and (d) elemental maps corresponding to the oxygen and titanium signal distribution, respectively.

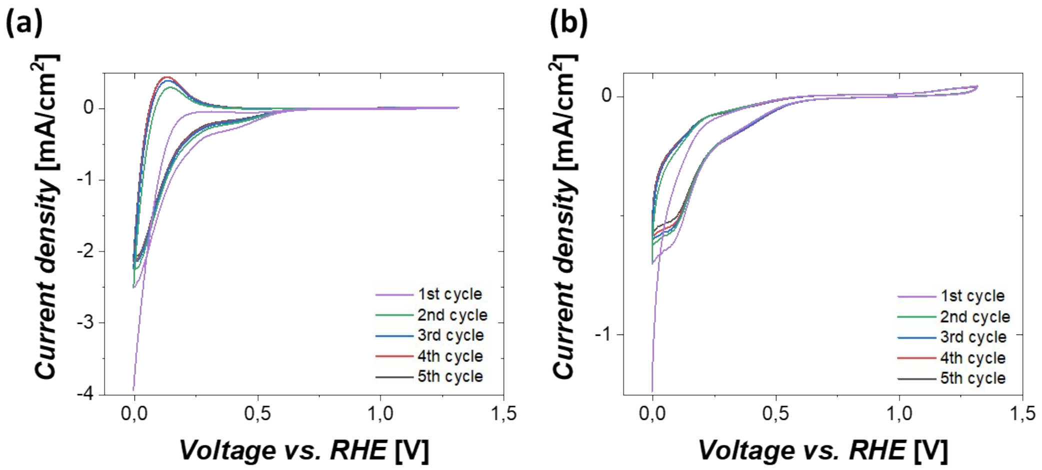

Figure S5. Initial cyclic voltammetry cycles for (a) Pt/Red and (b) Red/Pt.

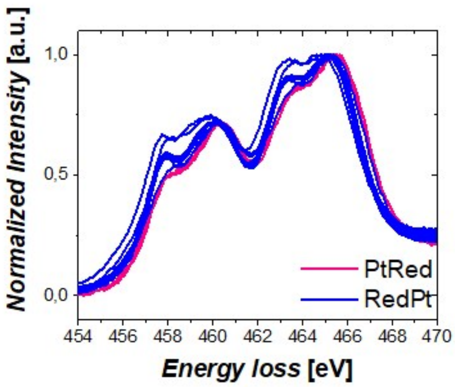

Figure S6. Averaged EELS spectrum showing Ti $L_{2,3}$ white lines of Pt/Red and Red/Pt nanobundles.
